# Near room temperature chemical vapor deposition of graphene with diluted methane and molten gallium catalyst

**DOI:** 10.1038/s41598-017-12380-w

**Published:** 2017-09-28

**Authors:** Jun-ichi Fujita, Takaki Hiyama, Ayaka Hirukawa, Takahiro Kondo, Junji Nakamura, Shin-ichi Ito, Ryosuke Araki, Yoshikazu Ito, Masaki Takeguchi, Woei Wu Pai

**Affiliations:** 1 Institute of Applied Physics, Graduate School of Pure and Applied Sciences, University of Tsukuba, 1-1-1 Tennodai, Tsukuba, Ibaraki 305-8573 Japan; 2 Faculty of Pure and Applied Sciences, University of Tsukuba, Tsukuba, Ibaraki 305-8573 Japan; 3 Tsukuba Research Center for Interdisciplinary Materials Science, University of Tsukuba, 1-1-1 Tennodai, Tsukuba, Ibaraki 305-8573 Japan; 4 National Institute for Materials Science, 1-2-1 Sengen, Tsukuba, Ibaraki 305-0047 Japan; 5 Center for Condensed Matter Sciences, National Taiwan University, Taipei, 106 Taiwan; 6 Department of Physics, National Taiwan University, Taipei, 106 Taiwan

## Abstract

Direct growth of graphene integrated into electronic devices is highly desirable but difficult due to the nominal ~1000 °C chemical vapor deposition (CVD) temperature, which can seriously deteriorate the substrates. Here we report a great reduction of graphene CVD temperature, down to 50 °C on sapphire and 100 °C on polycarbonate, by using dilute methane as the source and molten gallium (Ga) as catalysts. The very low temperature graphene synthesis is made possible by carbon attachment to the island edges of pre-existing graphene nuclei islands, and causes no damages to the substrates. A key benefit of using molten Ga catalyst is the enhanced methane absorption in Ga at lower temperatures; this leads to a surprisingly low apparent reaction barrier of ~0.16 eV below 300 °C. The faster growth kinetics due to a low reaction barrier and a demonstrated low-temperature graphene nuclei transfer protocol can facilitate practical direct graphene synthesis on many kinds of substrates down to 50–100 °C. Our results represent a significant progress in reducing graphene synthesis temperature and understanding its mechanism.

## Introduction

The reduction of graphene synthesis temperature remains a critical challenge for its application in electronic devices^1–4^. For example, the upper temperature limit to integrate graphene and Si-based devices should not exceed ~400 °C. It is further lowered to ~100 °C for plastic-based semiconducting electronic devices with organic molecules^5,6^. A method to directly grow good quality continuous graphene on versatile substrates without a graphene transfer step is also important. Established graphene transfer methods using PMMA with substrate removal etching and electrochemical delamination^7–9^ are cumbersome and costly for scaled-up production; contaminants (water, solvent, resists) incorporated and morphological irregularities (bumps, folds, wrinkles) introduced during transfer can deteriorate device performance. To overcome the above two major challenges, we present a new approach to grow continuous good quality graphene directly on versatile substrates down to 50 to 100 °C using diluted methane source and molten gallium catalyst. This represents an important step toward graphene synthesis and its integration into electronic devices.

The conventional chemical vapor deposition (CVD) methods on copper or nickel^10–14^ produce good-quality graphene with a high processing temperature of 1000 °C or more, and the use of graphene requires a transfer method. There have been attempts to reduce the graphene synthesis temperature. Such growth attempts require the production and migration of the atomic carbon species at a lower temperature. For example, low pressure chemical vapor deposition (LPCVD) with a unique choice of the carbon source such as benzene^15^, toluene^16^, methanol, ethanol, and propanol precursors^17^ was developed. Microwave and/or plasma assisted CVD is also a possible way to activate the source decomposition at 400~600 °C^18,19^. Introduction of nuclei seeds such as coronene^20^ and polycyclic aromatic hydrocarbon^21^ seems to reduce the growth temperature blow 300 °C. However, the quality of such low-temperature synthesized graphene generally need further improvement. In particular, the reduction of graphene synthesis temperature down to 100 °C and below would be crucial for integrated processing of graphene into future plastic and bio-electric devices, and yet has not been realized.

Recently, gallium, as a metal catalyst, has shown similar metallurgical characteristics for graphitization on the surface of liquid metals as copper^22–32^. For example, Ga vapor is an effective catalyst in graphene nanoribbon synthesis from an amyloid fibrils template, and the width of nanoribbon is controlled only by the width of pristine template^26^. Large-area graphene can be synthesized with methane CVD combining Ga vapor^28,29^. Monolayer graphene can also be readily produced on the Ga surface since the solubility of carbon in Ga is negligible^22,31^. More importantly, Ga is a liquid at room temperature. This fluidity of Ga presents several unique advantages as a graphene catalyst. First, molten Ga enhances the transport of carbon atoms and improves graphene growth kinetics; this helps reduce the graphene synthesis temperature. Second, molten Ga forms a conformal fluid-like interface with a substrate. This alleviates substrate lattice mismatch issue^30–32^. Moreover, a liquid catalyst could potentially enable low-temperature graphene CVD on not just flat planner substrates but also three-dimensional (3D) objects. Such versatile 3D graphene or composite structures are for example very attractive electrode materials for battery or supercapacitors^33–39^ and possibly for future plastic electronics and energy-related applications. Finally, molten Ga catalyst, unlike Cu or Ni, is easily removed by a gas jet after graphene growth. These advantages warrant detailed studies of graphene synthesis using molten Ga. Such studies should address not just how Ga catalyzes graphene growth but also whether Ga enables graphene growth at a lower temperature.

The graphene synthesis with molten gallium catalyst can remarkably produce continuous good-quality graphene on either sapphire or plastic polycarbonate substrate down to either 50 or 100 °C respectively. The key requirement for such low-temperature growth is to have pre-existing graphene island nuclei on the substrate. The nuclei are either produced by conventional CVD or by a special nuclei transfer technique at low temperature desribed below. Raman spectroscopy analyses indicate that carbon atoms in molten gallium are efficiently transported to pre-existing graphene nuclei, resulting in exclusive graphene growth at the graphene island edges. The remarkable low graphene synthesis temperature is facilitated by a surprisingly low apparent reaction barrier, which derived from Arrhenius plots of graphene growth and methane dehydrogenation rates gives a value of ~0.58 eV above 500 °C but a mere 0.16 eV below 300 °C. The very low barrier is a result of competing pathways, i.e., methane decomposition at the Ga surface and methane absorption in bulk liquid Ga followed by its decomposition in Ga; these two processes are respectively favored at higher and lower temperatures, thus explaining the weak temperature dependence and the unexpected low barrier. The pathway of methane absorption seems unique for molten Ga and is ineffective for other common graphene catalysts such as Cu, Ni.

The demonstration of our near-room-temperature graphene growth method and its successful application on, e.g., polycarbonate suggests its potential for future developments and opens up new possibilities for future integration of graphene into various, including plastic, electronic devices.

## Results

### Synthesis method

**Figure 1:**
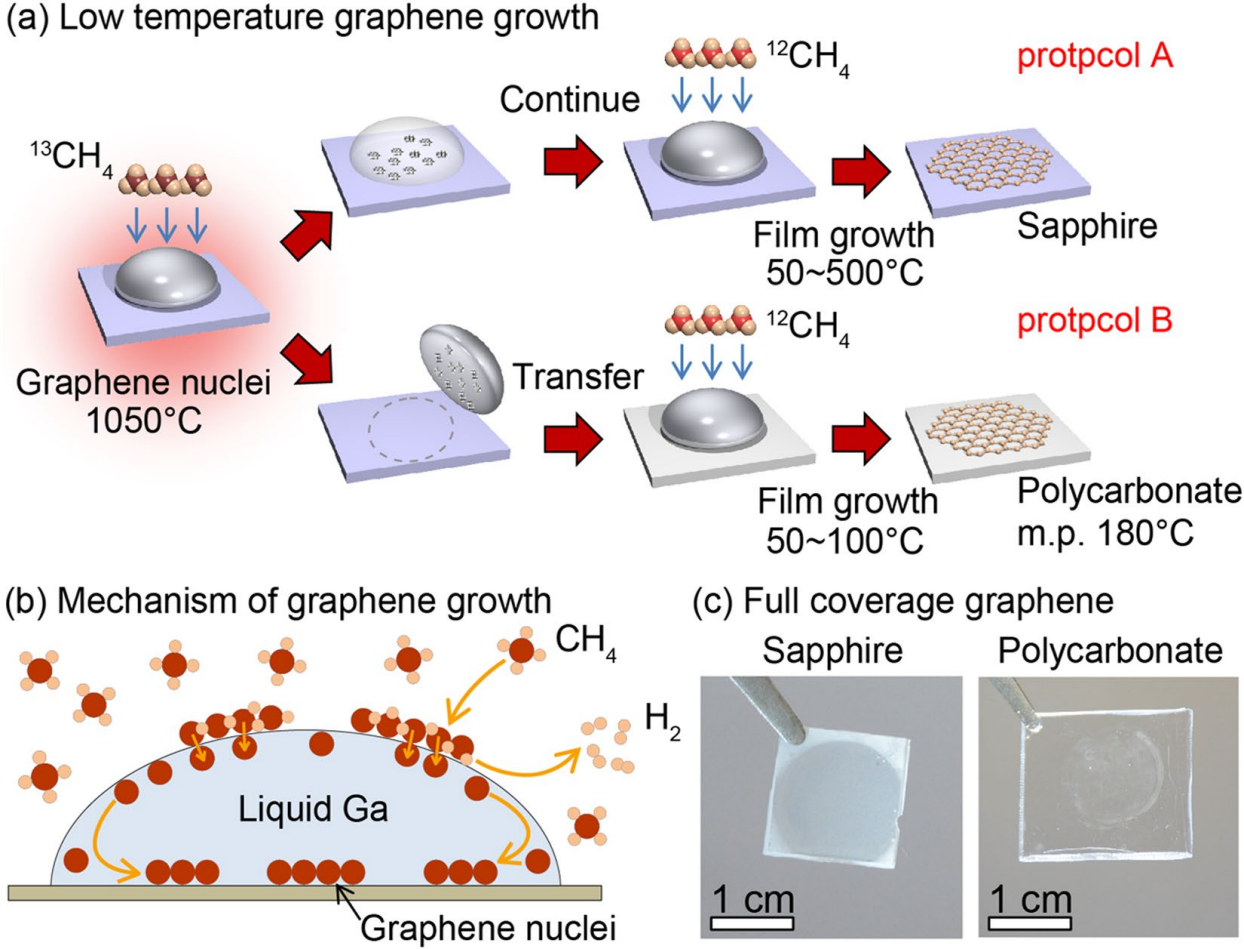
(**a**) Schematic illustration of two experimental s (A,B) for the low-temperature graphene edge growth using a molten liquid metal such as Ga and InGa. The first step is the formation of graphene nuclei using ^13^C-methane at 1050 °C for 300 sec. Lower-room temperature edge growth proceeded subsequently with ^12^CH_4_. See main text for details of both protocols. (**b**) Mechanism of graphene nuclei generation and film growth. (**c**) Optical images of full coverage graphene films on sapphire and polycarbonate substrates.

Our CVD source gas is diluted methane which consists of 1 sccm of 5% methane (diluted by 6N-Ar) mixed with 250 sccm of 6N-Ar. During the CVD growth, the source gas pressure was ~20 Pa. The substrate was C-face sapphire with a miscut angle of 0.2° or polycarbonate. Figure 1a illustrates two experimental protocols for graphene growth using molten Ga catalyst. In protocol A, graphene nuclei were first prepared with ^13^C-methane and molten Ga for 300 seconds at 1050 °C, followed by quenching of the substrate temperature. Detailed temperature profile for nuclei growth is shown in Fig. S1a. The used molten Ga was then removed by blowing N_2_ gas and replaced by a fresh Ga droplet. ^12^C-methane was then used for further graphene growth at various selected lower temperatures (Figure S1(b)). Using ^13^C- and ^12^C-methane separately in the nucleation and growth stages allowed us to isotope label the growth front of graphene^40^ and, in the present case, to demonstrate graphene growth proceeds at the nuclei island edges (termed edge-growth mode hereafter). Conversely, ^12^C- and ^13^C-methane separately used in the nucleation and growth was also tested (Figure S2) to support that edge-growth mode.

The presence of pre-existing graphene nuclei is a key requirement for subsequent low-temperature graphene edge growth. In addition to the nuclei preparation with conventional CVD in protocol A, protocol B employs a graphene nuclei transfer method across the solid-liquid phase transition of a graphene-enriched Ga droplet. This facilitates a whole graphene synthesis all at lower temperatures, and is successfully applied to the polycarbonate substrate.

### ^12^C/^13^C Raman mapping and spectra

**Figure 2:**
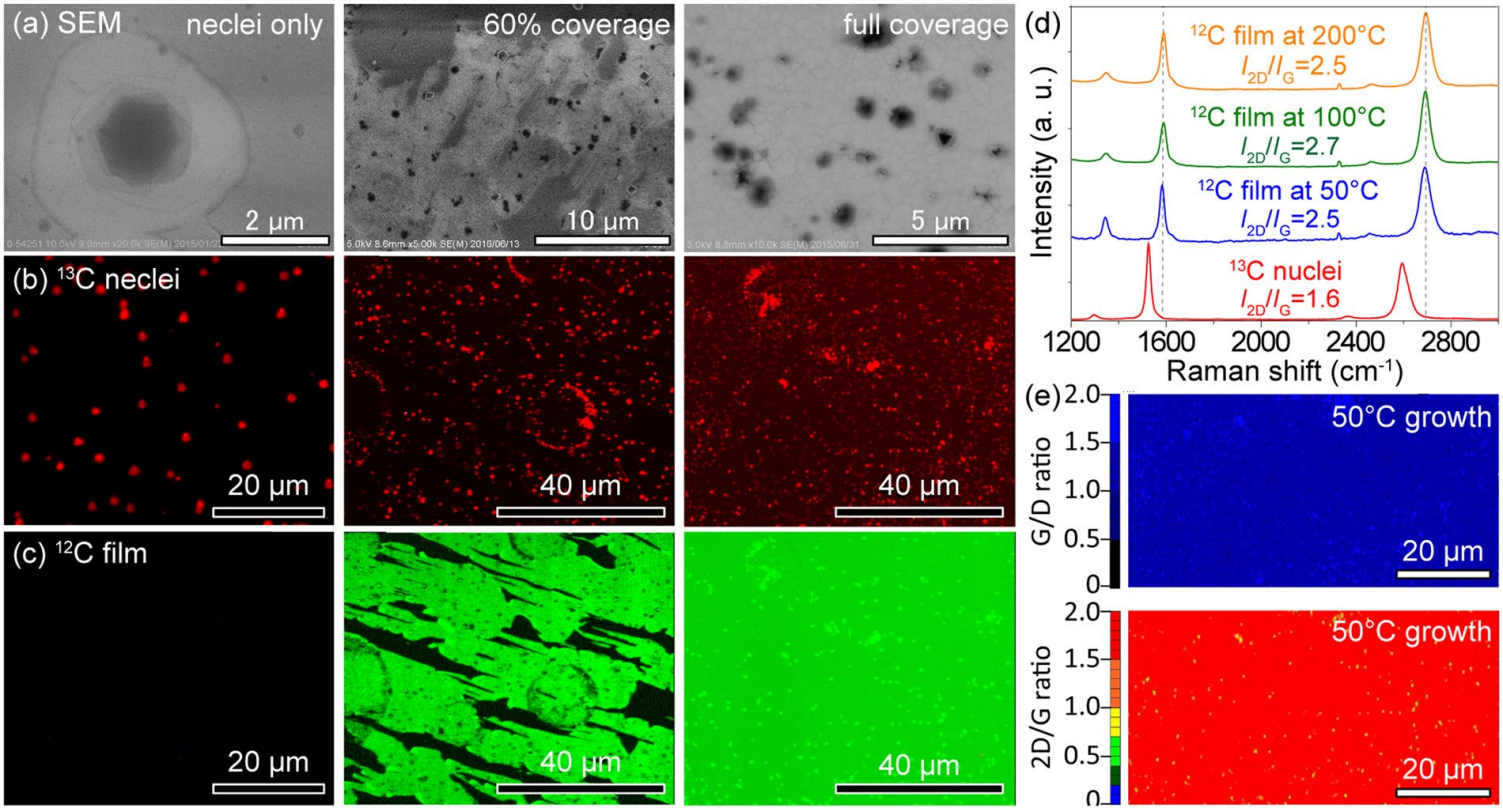
Raman spectroscopy of low temperature grown graphene. (**a**) SEM micrographs of graphene grown at 100 °C with different coverage; 0% (^13^C nuclei only), 60% (partly covered) and 100% (full coverage). (**b**,**c**) Raman G-band mapping of ^13^C- and ^12^C-graphene respectively at the coverages shown in (**a**). (**d**) Raman spectra of ^13^C graphene nuclei and ^12^C graphene films grown at 50, 100, and 200 °C. (**e**) Raman mapping of the *I*_G_/*I*_D_ and *I*_2D_/*I*_G_ intensity ratios on the 50 °C-grown sample.

The graphene nuclei prepared with ^13^C-methane at high temperature, after further low-temperature growth with ^12^C-methane, still consist of ^13^C only. The Raman maps and spectra for graphene nuclei to full coverage graphene film are illustrated in Fig. 2. The graphene nuclei typically grow in hexagonal spiral structures^28^ of ~0.5 to ~1 μm in diameter (Fig. 2a, Figs S3–S4). The quality of graphene nuclei is high, with a ~0.10 intensity ratio for the D and G bands (*I*_D_/*I*_G_). The edge growth temperature was 100 °C and the growth duration was 17 hours. At 300 °C, the whole growth duration was 2–3 hours; at 50 °C, 20 hours. The lowest onset temperature of graphene film growth from the nuclei edge was 50 °C.

In the Raman mapping, signals of the ^13^C-graphene nuclei and ^12^C-graphene film are separated. Despite the small size (~μm^2^) and low density (~10^4^/mm^2^) of graphene nuclei, nuclei are crucial sites that enable subsequent seamless and continual graphene growth around them. Graphene growth from the edge of an individual nucleus is isotropic but the overall morphology of edge-grown graphene can be affected by substrate step direction and appeared anisotropic. In the 60% coverage sample (Fig. 2a–c), some graphene nuclei tended to align with a step or step bunch. We frequently observed elongated graphene islands on a slightly inclined substrate (Fig. S4). Such anisotropic growth manner suggests that the substrate miscut or, more generally, substrate patterns, can be used to manipulate graphene growth morphology.

The quality of graphene films (Fig. 2d, Figs S5–S6) grown with ^13^C-graphene nuclei were investigated with different growth temperatures from 50 to 500 °C. The *I*_D_/*I*_G_ intensity ratio of the films were 0.51, 0.27, 0.25, 0.16, 0.26 for 50 °C, 100 °C, 200 °C, 300 °C, and 500 °C; for *I*_2D_/*I*_G_, 2.5, 2.7, 2.5, 2.3, 2.8 for 50 °C, 100 °C, 200 °C, 300 °C, and 500 °C. This shows that the defect density is low and not strongly influenced by the growth temperature. The spiral graphene nuclei prepared at 1050 °C are less defected, show a lower *I*_D_/*I*_G_ ratio, and tend to be multi-layer. Other low-temperature edge-growth regions tend to be monolayer but with slightly more defects. However, even on a film with the lowest growth temperature of 50 °C, the *I*_G_/*I*_D_ and *I*_2D_/*I*_G_ maps (Fig. 2e) indicate a very uniform and high-quality film. Most areas in an 80-μm square show a *I*_G_/*I*_D_ ratio ~1.5 and a *I*_2D_/*I*_G_ ratio ~2.0. Details of Raman spectra peak position, line width, and intensity ratio are summarized in Table S1.

### Atomic structure of low-temperature graphene films

**Figure 3:**
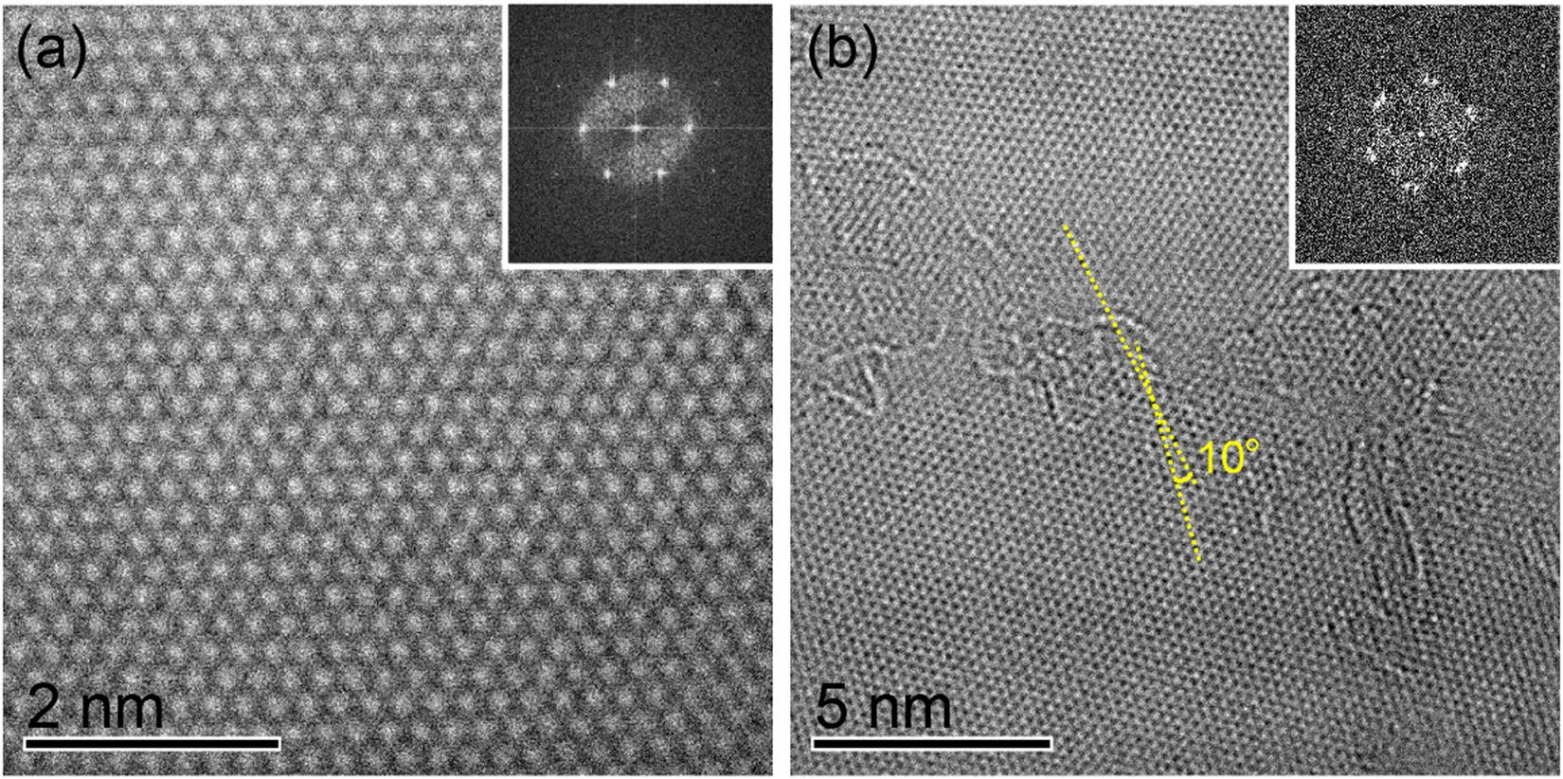
Atomic structures of low temperature grown graphene. (**a**) HR-TEM image of graphene film and its corresponding diffraction patterns. (**b**) HR-TEM image near the graphene grain boundary. The misoriented angle between graphene domains is within 10 degrees, and is seen as two adjacent diffraction patterns.

Perfect graphene lattice with hexagonal diffraction spots was observed for graphene film grown at 100 °C, as revealed by the high-resolution transmission electron microscopy (HR-TEM) images in Fig. 3a. Such a perfect lattice is beneficial for transport properties. Graphene domains do exist; near the grain boundary a <10 degrees misorientation is typical, as seen in many TEM images and diffraction patterns (Fig. 3b). Despite a 50% lattice mismatch between sapphire and graphene, graphene and nuclei do not appear in random orientation but are aligned within <10 degrees. The presence of the steps on our 0.2 degrees miscut C-faced sapphire substrate, having ~60 nm terrace width after recrystallization, can effectively align the nuclei orientation, as shown in Fig. S4. It is also noted that our grown graphene adheres well to the substrate and only shows wrinkles due to differential thermal contraction from the growth temperature to room temperature. The substrate step structure remains intact after growth and the catalytic reaction with molten gallium was rather gentle. This is discussed in Fig. S7.

### Importance of graphene nuclei in low-temperature graphene growth

The growth methods depicted in Fig. 1a involve initial preparation of graphene nuclei. To illustrate the essential roles of such pre-existing graphene nuclei, graphene growth at a lower temperature *T*_2_ was conducted directly without pre-existing nuclei. As shown in Fig. S8, reasonable graphene crystallinity is only possible when *T*_2_ is greater than 900 °C. At *T*_2_ = 1000 °C^28^, graphene with good quality is evident from well-separated D- and G-band Raman peaks, a small G-band FWHM (~25 cm^−1^), and a large *I*_2D_/*I*_G_ ratio of ~2 to 3. The quality of graphene quickly deteriorates if *T*_2_ is below 900 °C. No crystalline graphene can be identified at *T*_2_ < 600 °C regardless of a long synthesis time up to 24 hours. Therefore, the initial presence of graphene nuclei is essential – it greatly promotes graphene quality and growth speed while significantly reducing the growth temperature. To realize an all-at-low-temperature graphene synthesis, pre-existing graphene nuclei cannot be prepared with conventional CVD. We have devised a method that uses the liquid-solid phase transition of gallium (at ~30 °C) to transfer graphene nuclei between different substrates. After the transfer, it was observed that the graphene film grew only within the region where the Ga droplet was placed, not beyond the region. This phenomenon was also reported in the references ^27,30,32,41^. A gallium droplet, when solidified, can peel graphene nuclei off a sapphire substrate. The solidified Ga droplet then can be transferred to another substrate (e.g., polycarbonate). Upon melting, graphene nuclei embedded in the Ga droplet will be released onto the substrate.

### Migration path for carbon

We have demonstrated previously that the outermost surface of molten gallium can absorb up to 50 wt% of carbon in a ~5 nm thin skin layer^23^, despite a low carbon solubility in gallium. At ~1000 °C, carbon atoms diffusing through bulk liquid gallium is also possible^42^. In contrast to Cu-based graphene CVD, carbon retention and diffusion are therefore unlikely restricted to the Ga surface. If the gallium removal step after nuclei formation was skipped (Fig. 1a), we observed a randomly mixed ^12^C-^13^C graphene instead, as evidenced by the merging of pure ^12^C- and ^13^C-graphene Raman peaks^43^. The random mixture means that carbon atoms in Ga, once formed, do not immediately incorporate into graphene island edges. This delayed retention of carbon atoms inside Ga suggests carbon presence and transport in the near-surface region of molten Ga. Since the carbon production and transport are not restricted to the atomic interface between the molten Ga and substrate, the graphene growth kinetics is enhanced.

### Low-temperature graphene growth on polycarbonate

With the aforementioned graphene nuclei transfer method by solid-liquid phase transition of Ga, we demonstrated that graphene synthesis can be achieved on a plastic polycarbonate substrate (e.g., at 100 °C throughout). In Fig. S9, Raman map of graphene grown on polycarbonate at 100 °C shows well identified Raman spectra. Importantly, the graphene film synthesized on polycarbonate (Fig. 1c) also shows a full coverage.

### FET characteristics

**Figure 4:**
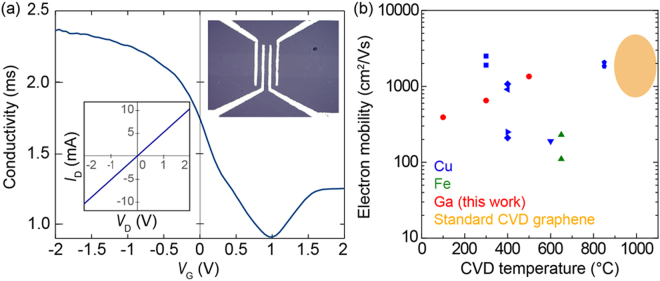
Graphene field-effect transistor performance. (**a**) Conductance curve of a FET made from graphene synthesized at 100 °C. A top-gated configuration with ionic liquid was used. (**b**) Dependence of FET electron mobility on the low growth temperature of graphene samples using Ga (red points) with references of graphene/Cu foils (blue rectangles, triangles, and diamonds) and graphene/Fe foil (green triangles)^15–21^. The orange circle presents the electron mobility range of graphene FET made from standard CVD graphene on Cu substrates.

The performance of field-effect transistors made from low-temperature growth graphene was investigated. A top gate configuration with an imidazolium-based ionic liquid (1-butyl-3-methylimidazolium hexafluorophosphate) was used^44^. The device and measured gated conductance curve are shown in Fig. 4a. All fabricated FET devices exhibited *p*-type characteristics, and the Dirac point at minimal channel conductance shifted to ~1 V gate bias. Hole mobility, estimated from the back gate voltage dependence of graphene conductivity, was ~390 cm^2^/Vs for graphene grown at 100 °C, ~650 cm^2^/Vs for graphene grown at 300 °C and ~1350 cm^2^/Vs for graphene grown at 500 °C. These mobility values rival that of conventional CVD graphene (Fig. 4b). Moreover, the higher mobility of graphene grown by the Ga based CVD method is also compared with the CVD graphene grown on Cu and Fe foils^15–21^. One notes that the mobility value of graphene grown at low temperatures of 100 °C was not reported previously. Considering the CVD graphene cannot be obtained below 900 °C with conventional CVD method using copper (Fig. S8) or below 100 °C with reported low-temperature CVD methods, the Ga based low temperature grown graphene film offers significant potential advantages in electronic device applications.

## Discussion

### Reaction barriers of graphene synthesis

The apparent reaction barrier for graphene growth is estimated from the temperature dependence of the mean average graphene growth speed [cm^2^/sec]. The mean growth speed was evaluated by the required time to reach a full graphene coverage of the interfacial area under a gallium droplet (0.8 cm in diameter and 0.5 cm^2^ in size). As the mean growth speeds at different temperatures were all evaluated when the graphene coverage just became full and the preparation procedure of nuclei was identical, the energy barriers for the rate-limiting steps can be estimated from the Arrhenius plot.

The Arrhenius plot shows two temperature windows with distinct energy barriers, i.e., a high-temperature region >500 °C with an activation barrier *E*_a_ of ~0.58 eV and a low-temperature region <300 °C with an activation barrier *E*_a_ of ~0.16 eV. The window between 300 °C to 500 °C is a transition range in which processes associated with the two above barrier values are both contributing. The *E*_a_ ~0.58 eV barrier is consistent with the reported values from 0.52 eV to 0.77 eV obtained with a Ni catalyst^45,46^. However, the observed low *E*_a_ ~0.16 eV for the low-temperature graphene edge growth is surprising. It suggests that a simple methane activation process is unlikely valid. Instead, parallel processes could be competing in the molten gallium catalyst, particularly at a lower temperature.

**Figure 5:**
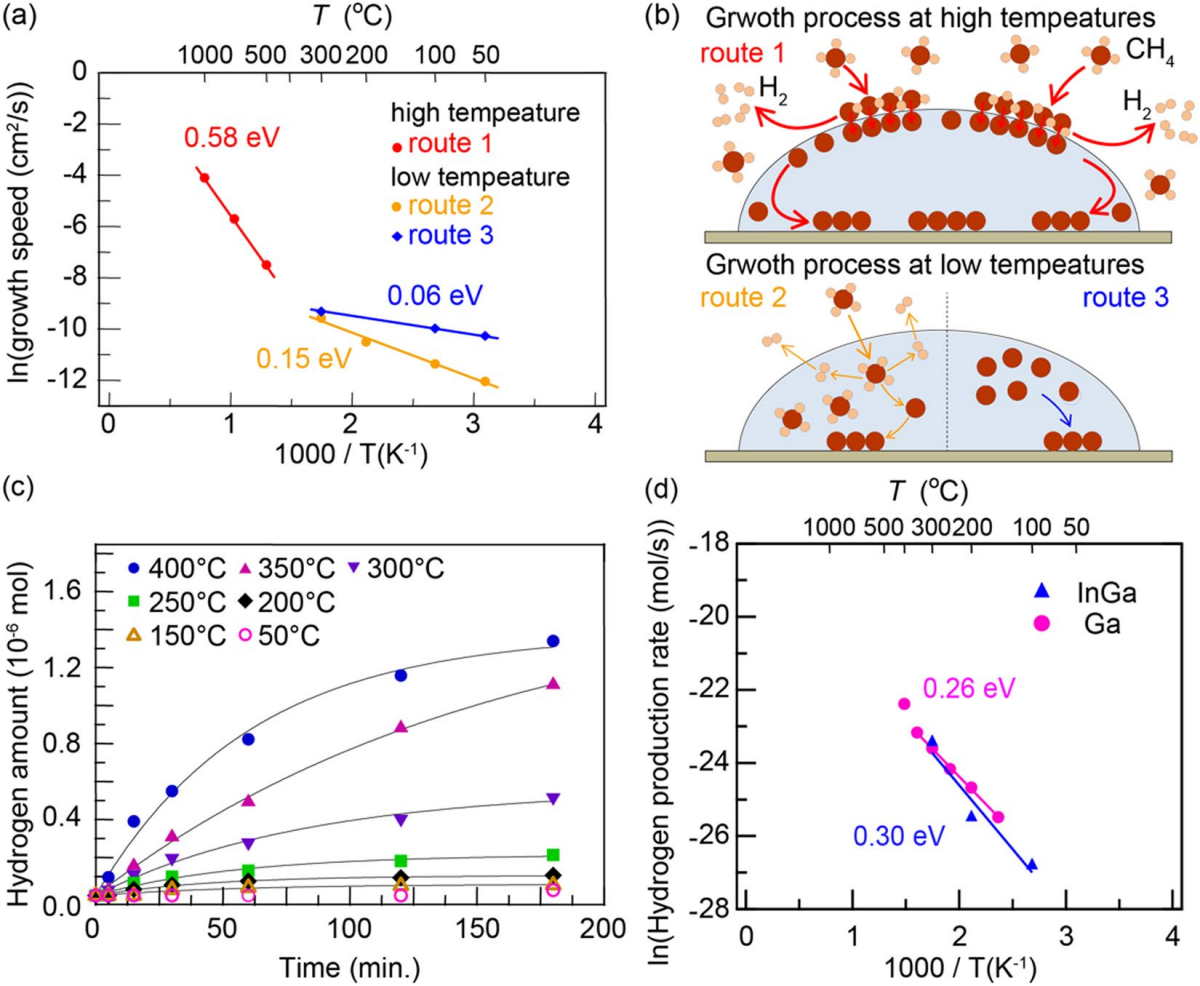
Catalytic properties of Ga for methane decomposition. (**a**) Arrhenius plot of the graphene growth speed at different conditions. The route 1 at high temperatures represents graphene growth via the methane decomposition on the Ga surface. The routes 2 and C at lower temperatures represent the graphene growth via the methane absorption into Ga followed by decomposition in Ga (route 2), and by residual carbon from decomposed methane in Ga (route 3). (**b**) An illustration of the respective growth mechanisms for routes 1, 2, and 3. (**c**) Time-dependent hydrogen production amount for methane decomposition in Ga at reaction temperatures of 50, 150, 200, 250, 300, 350, and 400 °C. The apparent barrier of hydrogen evolution is derived from the initial production rates. (**d**) Arrhenius plot of hydrogen production efficiency, for both Ga and InGa catalysts.

The effect of parallel processes can be illustrated by replacing fresh gallium droplets with carbon-enriched gallium droplets. Such C-enriched Ga drops can be obtained by sintering molten gallium at 1000 °C for 10 hours in a carbon crucible with diluted methane source gas to feed carbon atoms on the surface or in the Ga bulk. Using C-rich Ga remarkably reduces the required graphene growth time by about 40%, in particular at lower temperatures. The presence of background carbon atoms in C-enriched Ga not only enhances the graphene growth rate but also weakens the temperature dependence of carbon atom supply. The apparent barrier of graphene growth is further reduced to 0.06 eV (route 3 in Fig. 5a). At higher temperature, the differences between using fresh or C-enriched gallium become less significant. The methane activation rate provides a faster feeding of atomic carbons than that from C-enriched gallium droplets. At 500 °C, the slopes of the growth rates with or without C-enriched Ga converge.

### Absorption and decomposition of methane in molten gallium

We have measured the kinetics of methane decomposition into H_2_ and carbon atoms, CH_4_ → C + 2H_2_, by molten Ga catalysts. In these experiments, ~60 cc of atmospheric methane was kept in a reaction cell for 3 hours at various temperatures between 100 °C to 350 °C (see Method III). Hydrogen production by methane decomposition was then measured by gas chromatography. Figure 5c shows the hydrogen production rates; the hydrogen production with atmospheric methane is sublinear and saturates gradually with reaction time (explained in Figs S10 and S11). To obtain the apparent activation energy of methane decomposition (*E*_apparent_), a value of 0.26 eV is derived from the temperature-dependent initial slopes of the H_2_ production curves (Fig. 5d). The barrier of *E*_a_ ~0.26 eV appears to be rather small for methane decomposition. Typical transition metals, such as Fe and Ni, are known to catalyze methane decomposition at higher temperatures, for example, ~500 °C with iron-based binary alloys^47^. Additionally, the lowest temperature of methane decomposition is predicted to be ~300 °C on a copper surface^48^. The threshold temperature for the production of hydrogen on a transition metal is reported to be ~200 °C^49^.

We surmise that the observed low apparent barrier, without using C-enriched Ga droplets, is due to a new channel of feeding precursor CH_4_ into Ga. We propose a new mechanism to explain the unexpected small apparent activation energy. The mechanism involves the following reaction steps,

1 $${{\rm{CH}}}_{4}\rightleftharpoons {{\rm{CH}}}_{4({\rm{Ga}})}$$

methane absorption into bulk Ga

2 $${{\rm{CH}}}_{4({\rm{Ga}})}\to {{\rm{C}}}_{({\rm{Ga}})}+4{{\rm{H}}}_{({\rm{Ga}})}$$

decomposition of absorbed methane

3 $$4{{\rm{H}}}_{({\rm{Ga}})}\to 2{{\rm{H}}}_{2}$$

hydrogen gas production

4 $${{\rm{C}}}_{({\rm{Ga}})}\to {{\rm{C}}}_{({\rm{graphene}})}$$

graphene formationwhere CH_4(Ga)_, C_(Ga)_, and H_(Ga)_ stand for absorbed CH_4_, absorbed carbon atoms, and absorbed hydrogen atoms. Here, absorption of methane in Eq. (1) is the first step and the amount of CH_4_ in Ga is determined by the enthalpy Δ*H* of absorption, methane pressure, exposure time, and temperature. The Δ*H* of Eq. (1) is negative because the solubility of methane decreases as the temperature increases, as discussed in Fig. S11. The decomposition of absorbed methane in Eq. (2) is the rate-determining step for hydrogen production because the hydrogen recombination reaction is known to be fast compared with the rate of methane decomposition. With the above arguments, the apparent activation energy, *E*_apparent_, can be expressed by the following equation,

5 $${{E}}_{{\rm{a}}{\rm{p}}{\rm{p}}{\rm{a}}{\rm{r}}{\rm{e}}{\rm{n}}{\rm{t}}}={{E}}_{{\rm{d}}{\rm{e}}{\rm{c}}{\rm{o}}{\rm{m}}{\rm{p}}{\rm{o}}{\rm{s}}{\rm{i}}{\rm{t}}{\rm{i}}{\rm{o}}{\rm{n}}}+{\rm{\Delta }}{H}$$

here *E*_decomposition_ is the activation energy of methane decomposition inside Ga, Eq. (2). As a result of the negative value for Δ*H*, *E*_apparent_ is lower than *E*_decomposition_. A simple understanding of this lowering of apparent reaction barrier is the opposite temperature dependence of rates for methane absorption and decomposition (Eqs (1) and (2)) – this weakens the temperature dependence of the overall reaction rate.

Our proposed mechanism of methane decomposition in liquid Ga, which involves the decomposition of absorbed CH_4_ inside liquid Ga, explains the small activation energies of 0.16 eV for graphene growth rate (route 2, Fig. 5a) and the 0.26 eV for hydrogen evolution rate of methane (Fig. 5d). The difference of these two barriers may arise from details in the kinetics steps of Eqs (3) and (4). The proposed mechanism also explains the change in the apparent activation of graphene growth at 500 °C. Above 500 °C, decomposition of methane proceeds readily and mainly at the Ga surface (route 1, Fig. 5a) because CH_4_ absorption into bulk Ga becomes insignificant at higher temperatures. An illustration of the graphene growth of the routes 1, 2, and 3 (Fig. 5a) is shown in Fig. 5b.

To provide evidence of methane absorption in bulk Ga and its negative enthalpy, we have examined the absorption of methane and the decomposition of CH_4_ in Ga in more details. We found that methane was more favored to be absorbed at lower temperatures. Methane absorbed in bulk Ga at 50 °C till saturation is readily expelled from Ga when its temperature is raised to 350 °C (Fig. S11). In addition, the activation energy (*E*_decomposition_) of the decomposition of absorbed methane inside Ga is estimated to be 1.22 eV. We evaluated this barrier from hydrogen production rates measured at various temperatures below 350 °C after methane was first intentionally absorbed in Ga at a low temperature of 50 °C (See Fig. S12).

We emphasize that near-room-temperature graphene growth is impossible if there is no methane absorption in bulk liquid Ga. If one extrapolates the slope for CH_4_ decomposition at >500 °C to room temperature in Fig. 5a, the graphene growth rate will be negligibly small. Such an extrapolation from the Arrhenius plot is in contradiction to experiments - it suggests that a single mechanism of methane decomposition cannot account for both the high- and low-temperature graphene growth behavior. By noting that methane can be absorbed into liquid Ga, absorbed CH_4_ could prevail adsorbed CH_4_ in the low-temperature regime. In the <300 °C regime, a large number of methane molecules absorbed in the liquid Ga offsets the slower methane decomposition rate and lead to graphene growth at very low temperatures. At >500 °C, the CH_4_ saturates the Ga surface and decomposes quickly, and absorbed CH_4_ plays an insignificant role. To enhance the graphene growth at lower temperature, InGa catalyst was also tested. We have not noticed a significant difference as shown in Fig. 5d. However, searching other low-melting metal alloys to optimize catalytic activities is highly desirable.

In summary, we have firstly demonstrated that graphene can grow at near room temperature with molten gallium as an efficient catalyst under dilute methane atmosphere. Graphene grows at the edge of graphene island nuclei, as confirmed by isotope labeling with ^12^C- and ^13^C-methane in Raman spectroscopy. Such graphene nuclei are essential to enable low-temperature graphene growth. Molten gallium demonstrates high catalytic reactivity of carbon absorption and decomposition at lower temperatures (50–100 ^o^C); this gives a surprisingly small 0.16 eV barrier of graphene growth rate and 0.26 eV for hydrogen evolution rate. The fluidity of molten gallium contributes to easier transport and production of atomic carbon. The sequential combinations of methane absorption, methane dehydrogenation, and effective carbon transport through the molten gallium to graphene island edges underlie the driving forces for the near-room-temperature graphene CVD. Although graphene growth is confined to the interface of the Ga droplet and the substrate, the liquid nature of Ga catalyst is likely applicable to the fabrication of conformal graphene over 3D objects. The graphene FET devices show promising high electron mobility comparable to CVD graphene grown on Cu. Finally, we have tested an all-at-low-temperature graphene CVD on polycarbonate by transferring and seeding the essential graphene nuclei across the solid-liquid phase transition of Ga. This results in successful graphene growth on a plastic substrate at 100 °C for the first time, thus representing a significant step toward the integration of graphene in electronic devices, including the challenging flexible plastic electronic devices.

## Methods

### Evaluation for the synthesized grapheme

The crystal quality of the synthesized graphene was evaluated using Raman spectroscopy (RAMANplus, Nanophoton Corporation, Osaka, Japan and Renishaw) with a probe laser (λ = 532 nm), scanning electron microscopy (SEM, S-4800, Hitachi High-Technologies Corporation, Tokyo, Japan), and high-resolution transmission electron microscopy (TEM, JEM-ARM200F, JEOL Ltd, Tokyo, Japan). We used a KOH solution to separate graphene from the sapphire substrate, followed by the transfer process using polymethyl methacrylate (PMMA) onto a porous carbon mesh^7^ for TEM observation.

### Temperature and source gas feeding control for the nuclei growth

We used a C-face sapphire substrate (1 × 1 cm square) with a miscut angle of 0.2°. We recrystallized the sapphire surface at 1000 °C for 1 hour in ambient dry air to produce regular steps with ~40-nm-wide terraces. A Ga droplet with a diameter of ~5 to 8 mm was placed on the recrystallized substrate inserted in an one-inch diameter quartz tube reactor. Since graphene forms quickly at >1000 °C, quick temperature control was required for finer growth control and was achieved by using a furnace body-conveying technique. To quickly heat up the specimen from ~850 °C to ~1050 °C (in 3 minutes), the electric furnace heater body kept at 1050 °C was first placed ~10 cm away from the sample in a 250 sccm of Ar flow, and then was slid to the correct specimen position. To prepare graphene nuclei, diluted methane was let in the furnace for ~5 minutes at 1050 °C, and then the furnace body was again slid out to quench the nuclei growth. The specimen temperature was quickly cooled down to below 200 °C within 1 minute.

### Measurement of the methane decomposition and hydrogen production with molten gallium

We prepared gallium nanoparticles supported on SiO_2_ fibers for the measurement of methane dehydrogenation efficiency. The test specimen contained ~7.6 mg of gallium nanoparticles, which were dispersed on the fibers by temperature gradient heating between the gallium source and the fiber in a vacuum. The gallium was heated at 1000 °C in 10^−4^ Pa, and the fiber was ~10 cm away from the furnace center and was at ~400 °C.

The prepared sample of Ga nanoparticles (7.6 mg) supported on SiO_2_ fibers was then placed in a PYREX glass tube reaction cell with a volume of 57.40 cc. After evacuating the cell, the sample was heated up to a certain temperature (50, 150, 200, 250, 300, 350, or 400 °C). Then, CH_4_ (760 Torr) was introduced into the cell within 1 min. The production amount of H_2_ was evaluated immediately as a function of time by measuring 0.5 cc sampled gas species of by TCD gas chromatograph. The hydrogen amount for the y-axis of the Arrhenius plot (Fig. 5c) was for the total hydrogen amount produced in the reaction cell.

## Electronic supplementary material

Supplementary Information
