# Supplementary material for: Near room temperature chemical vapor deposition of graphene with diluted methane and molten gallium catalyst
Source: Sci Rep. 2017 Sep 28;7:12371. doi: 10.1038/s41598-017-12380-w (PMC5620074; doi:10.1038/s41598-017-12380-w)
Supplement: Supplementary file 1 — Supplementary Information [file 41598_2017_12380_MOESM1_ESM.pdf]

Electronic Supplementary Information

For

Near room temperature chemical vapor deposition of graphene with diluted methane and molten gallium catalyst

Jun-ichi Fujita,[1,3,] Takaki Hiyama,[1,3] Ayaka Hirukawa,[1,3], Takahiro Kondo,[2,3] Junji Nakamura,[2,3] Shin-ichi Ito,[2,3], Ryosuke Araki[1,3], Yoshikazu Ito[1], Masaki Takeguchi,[4] and Woei Wu Pai[5,6]*

[1]*Institute of Applied Physics, Graduate School of Pure and Applied Sciences, University of Tsukuba, 1-1-1 Tennodai, Tsukuba, Ibaraki 305-8573, Japan*

[2]*Faculty of Pure and Applied Sciences, University of Tsukuba, Tsukuba, Ibaraki 305-8573, Japan*

[3]*Tsukuba Research Center for Interdisciplinary Materials Science, University of Tsukuba, 1-1-1 Tennodai, Tsukuba, Ibaraki 305-8573, Japan*

[4]*National Institute for Materials Science, 1-2-1 Sengen, Tsukuba, Ibaraki 305-0047, Japan*

[5]*Center for Condensed Matter Sciences, National Taiwan University, Taipei 106, Taiwan*

[6]*Department of Physics, National Taiwan University, Taipei 106, Taiwan*

[*]Corresponding author

Jun-ichi Fujita

E-mail: fujita@bk.tsukuba.ac.jp

Address: Institute of Applied Physics, University of Tsukuba

1-1-1 Tennodai, Tsukuba 305-8573, Japan

Tel & Fax : +81-29-853-5302

Supplementary Information

Figure S1. Graphene synthesis temperature profile vs. time

Figure S1a depicts the temperature control for the graphene nuclei growth. The protocol of furnace temperature control is discussed in the main text Method II section. After graphene nucleation, molten Ga was removed by blowing N_2 gas and was replaced by a fresh Ga droplet. Subsequently, ^{12}C-methane was used for further graphene growth at various selected lower temperatures as shown in Fig. S1b.

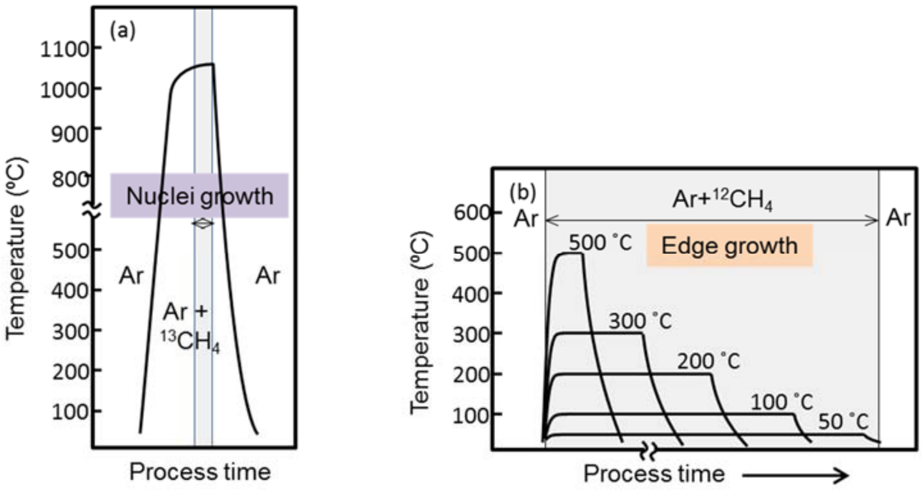

Figure S1(a) Temperature control diagram for the nuclei growth with $^{13}CH_4$. (b) Temperature control diagram for the low-temperature edge growth with $^{12}CH_4$.

Figure S2. SEM and Raman mapping graphene with C^{12} nuclei and C^{13} edge-grown film

Graphene synthesis with ^{12}C nuclei and subsequent ^{13}C edge-grown film. (a) SEM picture; (b) C^{12} graphene Raman mapping; (c) C^{13} graphene Raman mapping; (d) Raman spectra at selected points. The isotope labeling in both "C^{13}-nuclei/C^{12}-film" and the reverse "C^{12}-nuclei/C^{13}-film" case support the same edge-growth mechanism.

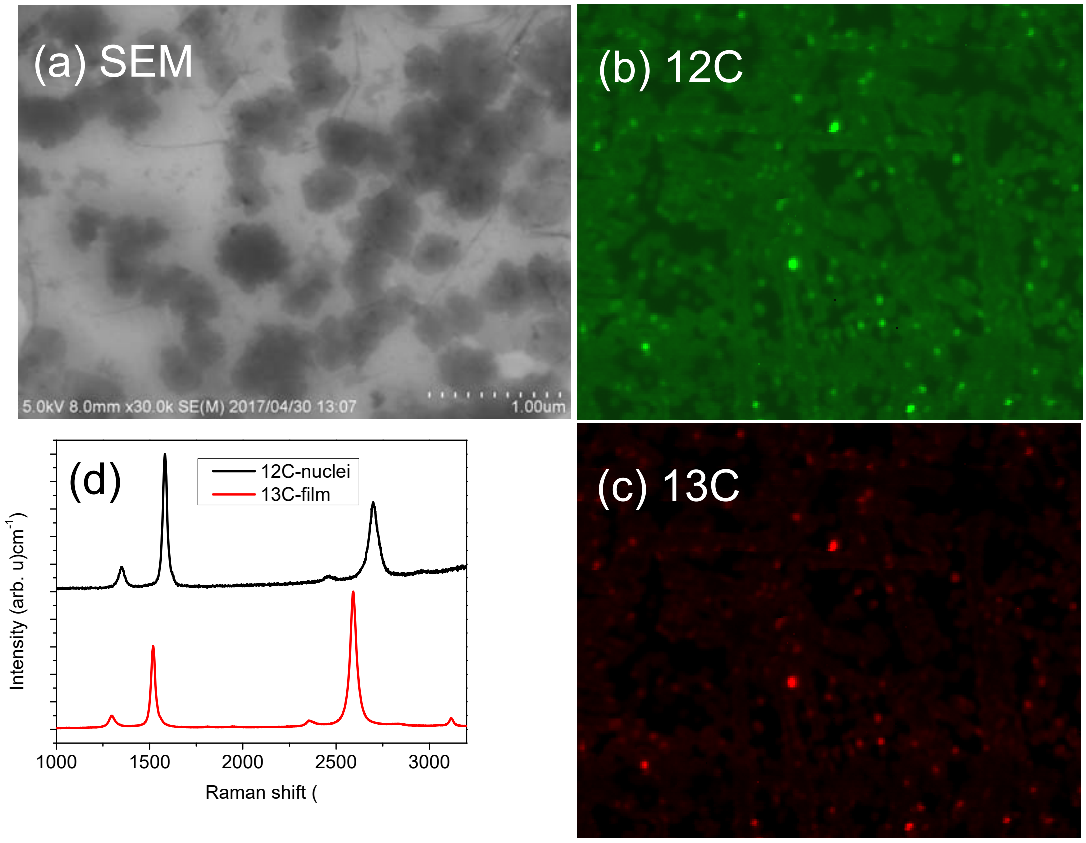

Figure S2. ^{12}C graphene nuclei and ^{13}C film. (a) SEM; (b) ^{12}C nuclei; (c) ^{13}C film; (d) Raman spectra.

Figure S3. Characterization of graphene nuclei

The graphene nuclei typically have a hexagon-shaped spiral structure and a diameter of ~0.5 to 1 μm. The white triangles, as seen in (a) and (b), are single-layered graphene nuclei exhibiting some wrinkles across the surface. In contrast, spiral nuclei are observed as dark colored hexagons since the electrical conductivity of the single layer of graphene nuclei was poor compared with the multi-layered spiral nuclei. While the nuclei edge exhibited a small misalignment to the step edge, many nuclei were roughly aligned with the sapphire step edge, as shown in (c). The spiral structure is also clearly seen in AFM friction image in (d).

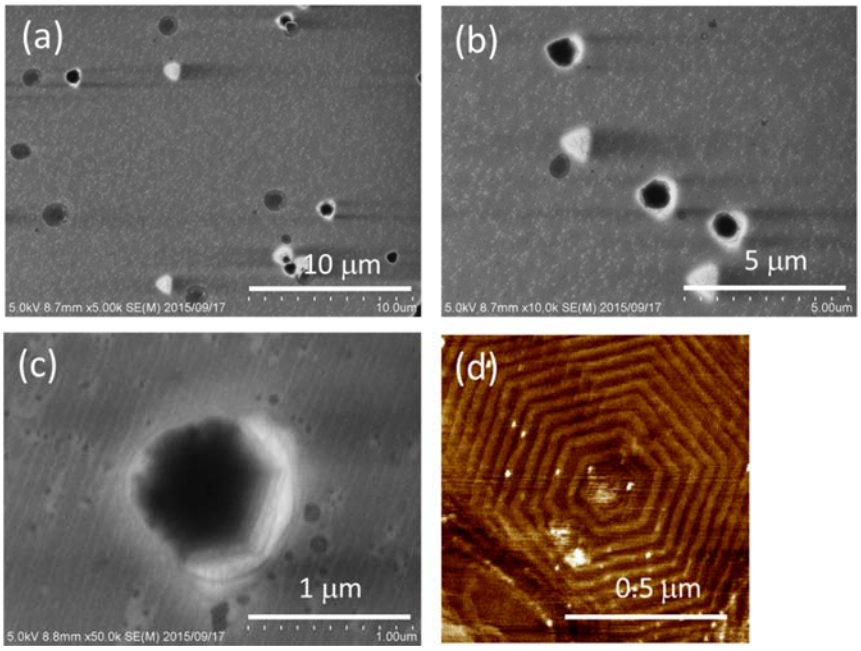

Figure S3. (a), (b) and (c) SEM images of graphene nuclei. (d) AFM friction image of a spiral nucleus.

Figure S4. Epitaxial alignment of nuclei, step edge, and graphene

The edge-growth graphene islands preferentially align with the step edge direction. Connected nuclei tend to progress along the step edge. As shown in (a) and (b), hexagonal shaped peripheral graphene edges are connected to each other. One side of the nuclei edge is roughly aligned to the step edge direction, as shown in (c). Schematics of the heteroepitaxial relationship between the sapphire c-face and graphene is illustrated, where <11-02>$_{sapphire}$ is aligned to <11-02>$_{graphene}$.

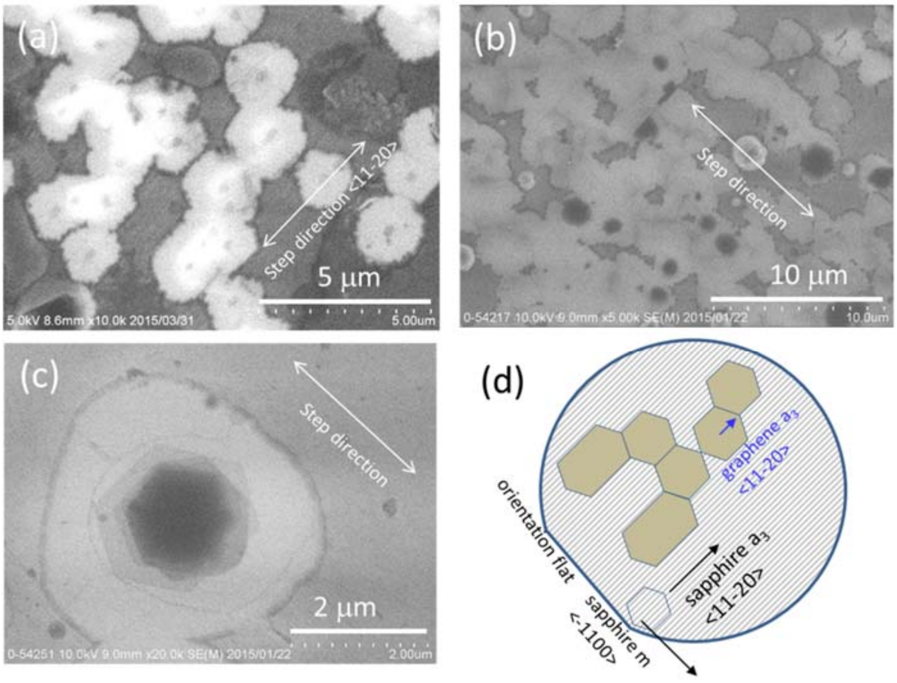

Figure S4. (a), (b) and (c) SEM images of the edge growth of graphene; (d) schematics of the heteroepitaxial relationship between the sapphire c-face and graphene.

Figure S5. Raman spectra of edge-grown graphene prepared at different temperatures.

Figure S5 shows a series of Raman spectra for samples with nearly full graphene coverage. Remarkably, the growth temperature can be as low as 50 °C, which is just above the melting point of gallium at 30 °C. All Raman spectra include signals from the ^{13}C-nuclei and ^{12}C-island regions, but the ^{12}C signal dominates. Although a defect- or disorder-induced D peak was observed at ~1340 cm^{-1} from 50 °C to 500 °C, its intensity was reasonably low and constant at >200 °C and only began to increase slightly at <200 °C. It is remarkable that the graphene quality hardly deteriorates even if the growth temperature changes from 500°C to 50°C.

Since the growth kinetics depends on growth temperature, it is noted that full coverage of graphene may require up to 30 hours of growth at 50 °C.

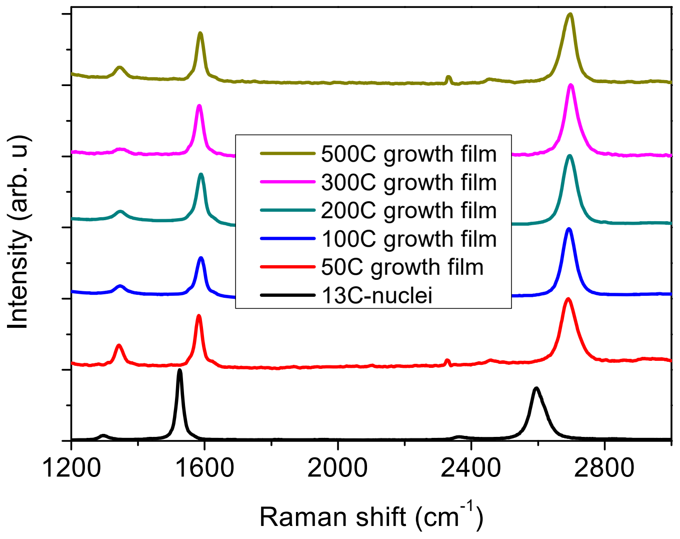

Figure S5 Raman spectra of ^{13}C graphene nuclei and the ^{12}C film grown at different temperatures.

Figure S6.   Raman spectra taken at ^{13}C nuclei and whole area

Raman mapping of ^{13}C- and ^{12}C-graphene, and spectra taken at ^{13}C graphene nuclei (red) and the whole mapped area (green) for graphene grown at 500 °C.

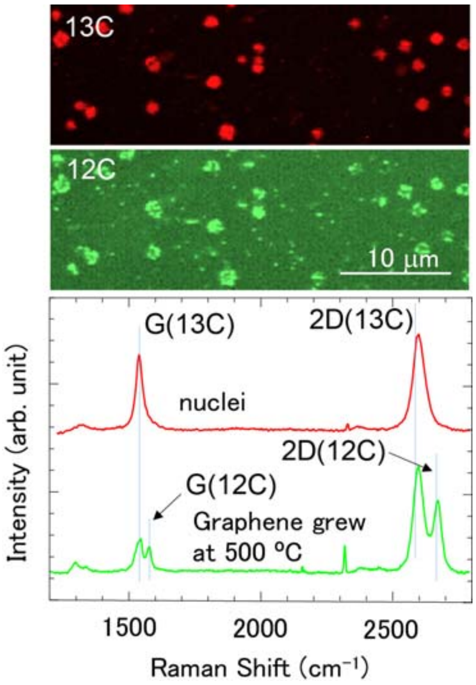

Figure S6.   Raman mapping of ^{13}C- and ^{12}C-graphene, and spectra.

Table S1. Summary of Raman spectra measurements

The peak position, line width, and intensity ratio of the graphene nuclei and films grown at different temperatures. Unit of the spectra and line width is cm^{-1}. Intensities were measured at the corresponding peak positions.

	D band<br>Line width	G band<br>Line width	D' band<br>Line width	2D band<br>Line width	I_D/I_G	I_{2D}/I_G
^{13}C graphene nuclei	1296<br>16	1525<br>11	1555<br>20	2597<br>23	0.10	1.55
^{12}C graphene film<br>50C	1345<br>15	1584<br>13	1622<br>10	2692<br>25	0.51	2.5
^{12}C graphene film<br>100C	1349<br>18	1590<br>14	1629<br>10	2694<br>21	0.27	2.7
^{12}C graphene film<br>200C	1349<br>18	1590<br>14	1630<br>11	2696<br>23	0.25	2.5
^{12}C graphene film<br>300C	1350<br>19	1585<br>13	1625<br>11	2700<br>21	0.16	2.3
^{12}C graphene film<br>500C	1347<br>14	1587<br>12	1620<br>14	2695<br>24	0.26	2.8
^{12}C graphene nuclei	1350<br>15	1583<br>14	1620<br>10	2700<br>25	0.17	1.1
^{13}C graphene film<br>100C	1298<br>18	1519<br>13	1550<br>20	2592<br>20	0.21	2.6

Figure S7 Wrinkles on graphene grown at different temperature

Bumps, folds, and wrinkles are commonly observed in transferred graphene. Even in CVD graphene before transfer, wrinkles are frequently observed, likely arising from the different thermal expansion of graphene and substrate and is hard to avoid. Our edge growth graphene also shows wrinkles. The wrinkle height is ~3 to <5 nm for graphene synthesized at 500°C and reduces to ~0.3 to <1.5 nm for graphene grown at 50°C and 100 °C. The observed height and its temperature dependence are remarkably consistent with a simple estimate. Thermal expansion coefficient is negative for graphene (-0.8×10^{-6}/K) and positive for sapphire (7×10^{-6} /K). With a mean wrinkle period of ~500 nm, an in-plane strain of 4×10^{-3} is estimated for graphene cooled from 500 °C to 30 °C. Experiments show that this strain is relaxed within a ~10 nm wrinkled region. A simple trigonometric calculation gives an expected wrinkle height of ~3 nm. Similarly, an in-plane strain of 2×10^{-4} is estimated for the 100 °C case and the estimated height of a 10-nm wide wrinkle are ~0.7 nm. Both of the above estimates agree well with the observed wrinkle heights, suggesting the grown graphene adheres well to sapphire, and the relaxation of strain from differential thermal expansion is confined in narrow wrinkled regions. While the grown graphene has wrinkles, the step structure of substrate remains intact after growth. The catalytic reaction with molten gallium at lower temperatures was relatively gentle. Such a "soft" catalytic reaction, which leaves the substrate's pristine features unchanged, is an important benefit for delicate substrates such as organic or patterned semiconductor surfaces.

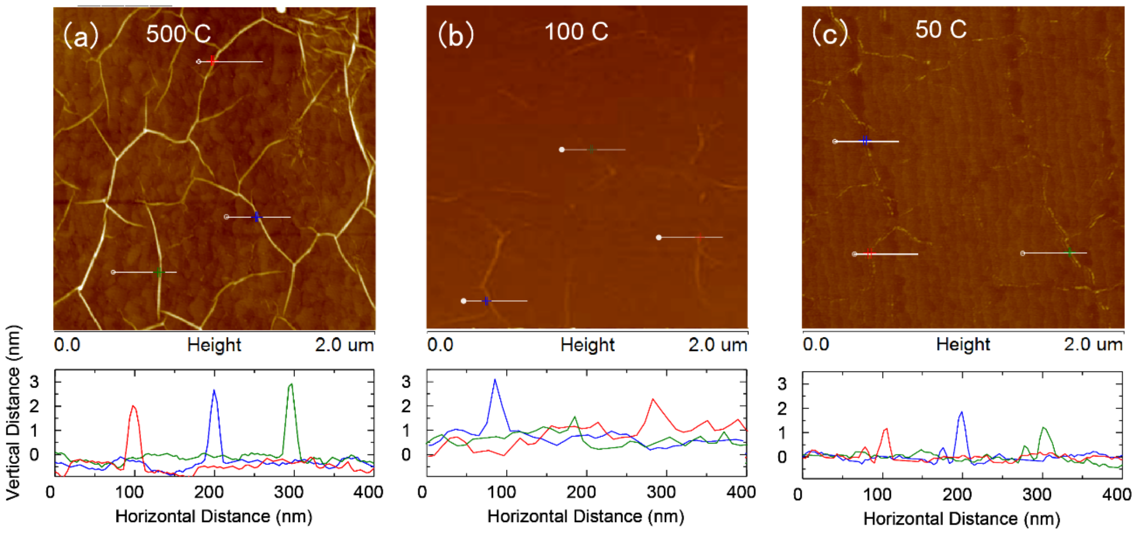

Figure S7. AFM image of graphene synthesized at 50°C, 100°C and 500°C and three cross-section profiles across wrinkles.

Figure S8 Raman spectra for graphene grown at different temperature, without pre-existing nuclei

Graphene growth is distinct with or without the graphene nuclei. The graphene synthesized at (a) 1000 °C without the nuclei still shows a clear peak separation of D and G, a small G peak FWHM of ~25 cm^{-1}. However, graphene growth at reduced temperature shows a clear increase of the D peak intensity (see (b)) and a strong suppression of the G peak intensity (see (c)). Further reduction of the growth temperature to < 800 °C produced a heavily disordered graphitic component rather than graphene, as shown in (d) and (e). No graphitic components in the Raman spectrum can be detected when the synthesis temperature was below (f) 500 °C

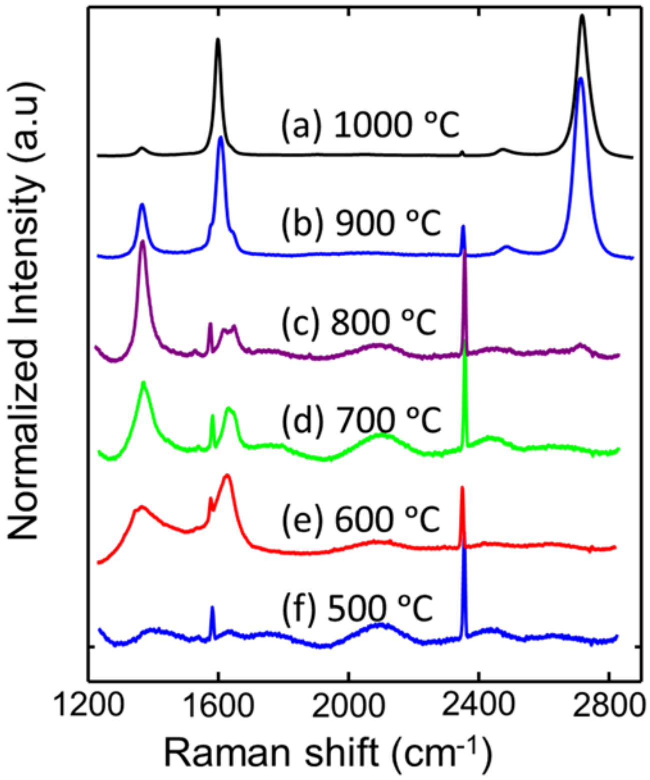

Figure S8. Raman spectra for graphene grown at different temperatures without the existence of graphene nuclei.

Figure S9 Raman map and spectra of graphene grown on a polycarbonate substrate

Raman mapping (80 µm x 20 µm) of the 2D/G ratio for graphene grown on polycarbonate at 100 °C is shown in in (a). Raman spectra at (i) bare polycarbonate, (ii) graphene nuclei (^{13}C; area #1), and (iii) edge-grown graphene (^{12}C; area #2) are shown in (b). Areas #1,#2 are shown as yellow rectangles. Since polycarbonate shows Raman peaks overlapped with the G and 2D bands of ^{12}C graphene, we use ^{13}C to label the graphene growth. We first used a gallium droplet to prepare ^{13}C graphene nuclei and then used the same gallium droplet to deposit ^{12}C in the edge-growth step. Due to the delayed retention of carbon in Ga, graphene grown on the polycarbonate is expected to show Raman signals of mixed ^{12}C- and ^{13}C-graphene.

Since the G-band of mixed graphene is red shifted from the G peak of polycarbonate, the small splitting of G band in the curve (ii) comes from the ^{13}C nuclei and the mixed graphene. Also in curve (ii) a 2D peak stronger than that of polycarbonate is observed. In curve (iii), mixed and wider G and 2D peaks due to the solid solution of ^{13}C and ^{12}C are observed. Those results suggested that graphene is successfully grown beyond graphene nuclei. We thus conclude that graphene CVD is also possible on a plastic surface even at 100 °C.

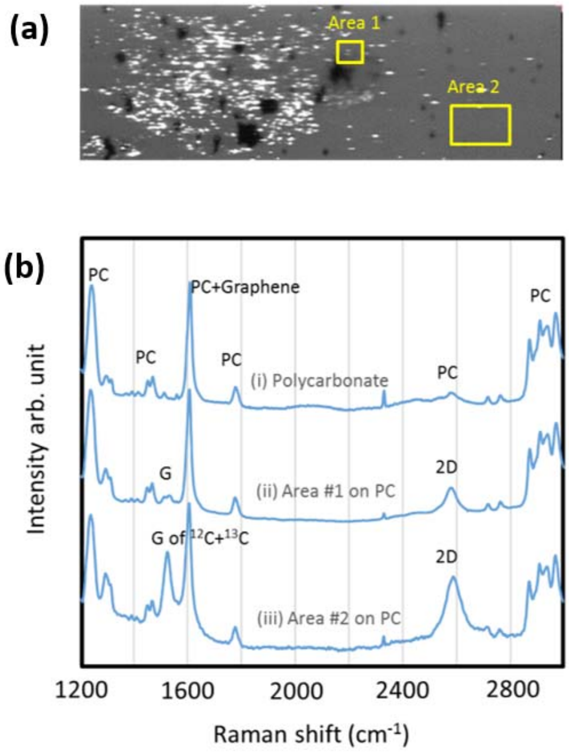

Figure S9. Raman map and spectra of graphene grown on a polycarbonate substrate at 100 °C.

Figure S10. Preparation of gallium nanoparticles for catalysis studies

The diameter of gallium nanoparticles dispersed on the SiO_2 fibers (~5 μm in diameter) varies from 10 nm to several hundred nanometers ((a),(b)). Carbon atoms were accumulated at the Ga droplet surface, and some carbon nanotube and nanofibers grew from the bottom of gallium nanoparticles ((c),(d)).

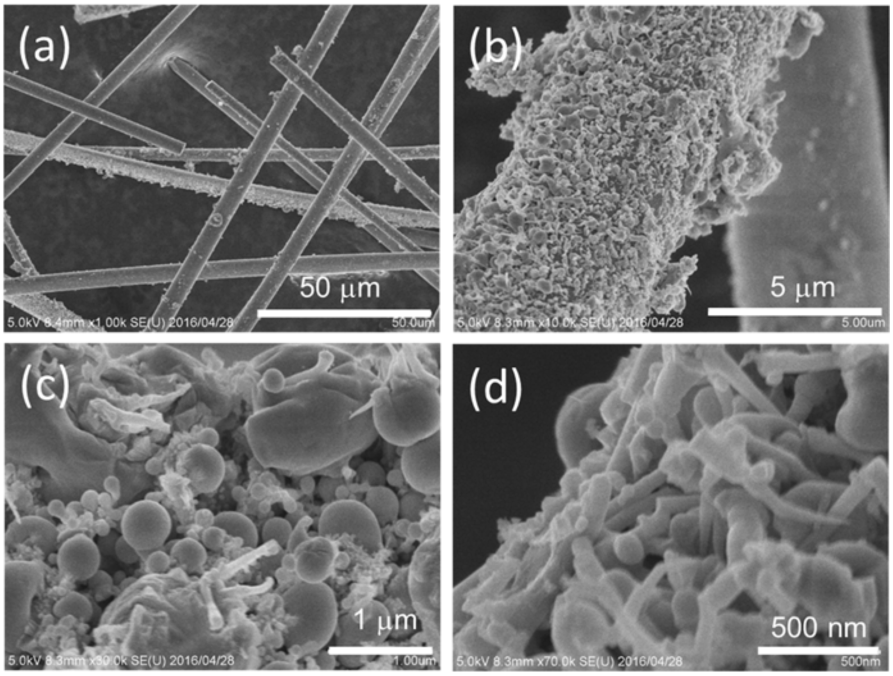

Figure S10. SEM images of gallium nanoparticles embedded on SiO_2 fibers.

Figure S11. Methane absorption in Ga and its release and decomposition upon heating

We have examined the absorption and decomposition of CH_4 in Ga. Here, CH_4 was absorbed in Ga at a low temperature of 50 °C followed by evacuation of gaseous CH_4. Absorbed CH_4 in Ga and dissociated CH_4 in Ga were then examined by measuring the production amount of CH_4 and H_2 at 350 °C in Ar atmosphere. More specifically, a sample of Ga (7.6 mg) supported on SiO_2 fiber was first placed into a PYREX glass tube reaction cell with a volume of 57.40 cc. After evacuating the cell, CH_4 (760 Torr) was introduced at 50 °C into the cell. After a certain exposure time (15, 30, 60, 120, and 180 minutes), CH_4 was evacuated within one minute at 50 °C, and then Ar (760 Torr) was introduced into the cell. Subsequently, the cell temperature was raised up to 350 °C in 10 minutes, and the production of CH_4 and H_2 was evaluated as a function of time by measuring the sampled gas species of 0.5 cc by TCD gas chromatograph, as shown in (a)-(e). As summarized in (f), produced CH_4 and H_2 from Ga were detected. It shows that CH_4 absorbed into Ga at 50 °C was expelled out of Ga at 350 °C, and a part of the absorbed CH_4 was decomposed during the process. The black dash line is the total absorbed CH_4 amount, which saturates after ~1 hr.

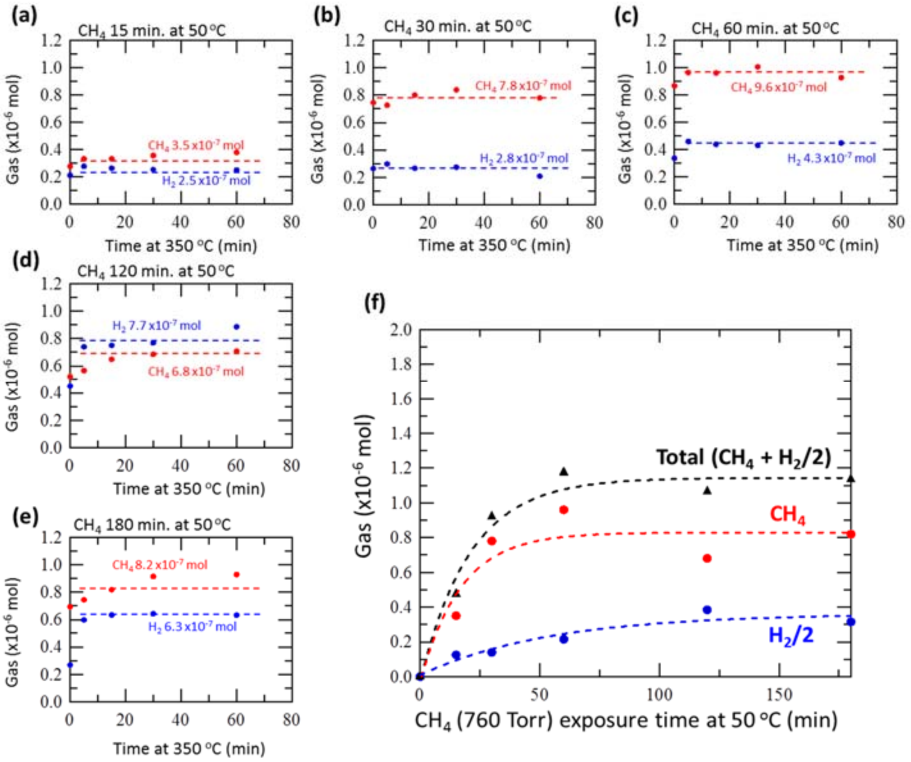

Figure S11. Absorption and decomposition of CH_4 in molten gallium.

Figure S12. Decomposition of absorbed methane in molten gallium: temperature dependence and barrier

We have examined the decomposition of methane inside Ga, as a function of temperature. Here, CH_4 was absorbed in Ga at a low temperature of 50 ^{o}C followed by evacuation of gaseous CH_4. Decomposition of absorbed CH_4 in Ga was then examined by measuring the production amount of CH_4 and H_2 at various temperatures in Ar atmosphere. More specifically, the sample of Ga (7.6 mg) supported on SiO_2 fiber was first placed into a PYREX glass tube reaction cell with a volume of 57.40 cc. After the evacuation in the cell, CH_4 (760 Torr) was introduced at 50 °C into the cell. After 180 min. of exposure in atmospheric methane, CH_4 was evacuated 1 min. at 50 °C and then Ar (760 Torr) was introduced into the cell. Subsequently, the cell temperature was increased up to various temperatures (100 °C, 150 °C, 200 °C, 250 °C, 275 °C, 300 °C, 325 °C, and 350 °C) and the production amount of CH_4 and H_2 was evaluated as a function of time by measuring 0.5 cc sampled gas species by TCD gas chromatograph as shown in (a)-(h). Here, we plot the total gas amount produced in the reaction cell (multiplying the sampled gas amount by 57.4 (cc)/ 0.5 (cc)). However, H_2 production is too low to give reliable estimates in Figs. (f)-(h). The Arrhenius plot for hydrogen production rate, as shown in (i), was derived from the initial slopes of the hydrogen uptake curves as shown by dashed lines in (a)-(e). From the slope in (i), the activation energy is estimated as 1.22 eV for the decomposition of absorbed methane in Ga.

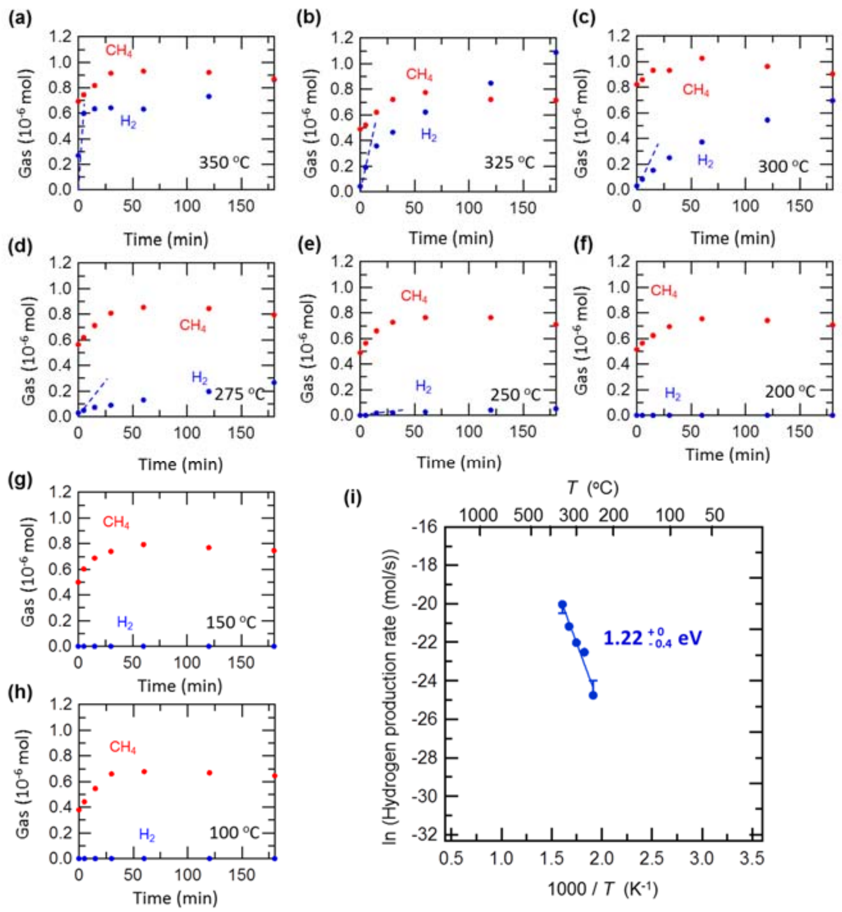

Figure S12. Temperature dependence of decomposition of absorbed CH_4 in molten gallium.
